# Qing-Xin-Jie-Yu Granule attenuates myocardial infarction-induced inflammatory response by regulating the MK2/TTP pathway

**DOI:** 10.1080/13880209.2025.2467377

**Published:** 2025-02-21

**Authors:** Jianghan Qi, Xiaoyao Gao, Ying Han, Meiling Yang, Chenyi Wei, Ling Zhang, Jianfeng Chu

**Affiliations:** ^a^ College of Integrative Medicine, Academy of Integrative Medicine, Fujian University of Traditional Chinese Medicine, Fuzhou, Fujian, China; ^b^ Fujian Key Laboratory of Integrative Medicine on Geriatrics, Fujian University of Traditional Chinese Medicine, Fuzhou, Fujian, China; ^c^ Department of Acupuncture and Moxibustion, The Third Affiliated Hospital of Fujian University of Traditional Chinese Medicine, Fuzhou, Fujian, China

**Keywords:** Ischemic heart disease, traditional Chinese medicine formula, inflammation-induced injury

## Abstract

**Context:**

Qing-Xin-Jie-Yu Granule (QXJYG) has shown promise in the treatment of myocardial infarction. However, the mechanism of action of QXJYG underlying its anti-inflammation remain unknown.

**Objective:**

The study aimed to evaluate the effectiveness and mechanism of QXJYG in a mouse model of myocardial infarction and hypoxia-induced H9C2 cells.

**Materials and methods:**

Myocardial infarction was induced in mice *via* left anterior descending coronary artery ligation, and hypoxia-induced H9C2 cells was served as the *in vitro* model. The cardiac function was evaluated by echocardiography, while myocardial tissue pathology was examined using HE and Masson’s trichrome staining. Changes in serum markers of cardiac injury were measured using ELISA kits. The levels of inflammatory cytokines in both the serum and cardiac tissue were quantified using the Bio-Plex Pro Mouse Chemokine assay, and hypoxia-induced inflammatory factors in H9C2 cells were assessed by RT-qPCR. Additionally, western blot analysis was conducted to evaluate the expression of proteins related to the MK2/TTP signaling pathway both *in vivo* and *in vitro* experiments.

**Results:**

QXJYG significantly enhanced cardiac function in mice with myocardial infarction, as evidenced by improved myocardial tissue structure, reduced collagen fiber deposition, and lowered serum levels of creatine kinase isoenzyme MB (CK-MB), cardiac Troponin T (cTnT), and brain Natriuretic Peptide (BNP). QXJYG may reduce the expression of inflammatory factors in both the heart and serum of myocardial infarction-induced mice and attenuate hypoxia-induced levels of inflammatory factors in cardiomyocytes by decreasing the ratio of p-MK2/MK2 and increasing the protein expression of TTP.

**Discussion and conclusions:**

QXJYG improved cardiac function and reduced injury, fibrosis, and inflammation after myocardial infarction, likely through modulation of the MK2/TTP signaling pathway.

## Introduction

In 2021, approximately 20.5 million people worldwide succumbed to cardiovascular disease, accounting for about one-third of the total global mortality (World Heart Report 2023: Confronting the World’s Number One Killer 2023). Among the spectrum of cardiovascular diseases, ischemic heart disease emerged as the leading cause of death due to its high fatality rate. Myocardial infarction, a manifestation of ischemic heart disease, occurs due to obstruction of coronary blood flow, resulting in localized or widespread necrosis of myocardial tissue. This process involves both programmed and non-programmed cell death of cardiomyocytes, contributing to extensive myocardial injury (Chang et al. 2024).

Following myocardial infarction, cardiac tissue reconstruction is initiated by an inflammatory response (Lv et al. 2021). During this phase, inflammatory cells clear necrotic cardiomyocytes and damage extracellular matrix *via* phagocytosis, followed by the activation of anti-inflammatory mechanisms to restore immune balance (Chang et al. 2023; Wang et al. 2018). However, prolonged inflammation, an exaggerated immune response, or impaired resolution of inflammation can exacerbate tissue damage and contribute to adverse ventricular remodeling (Frangogiannis, 2014) . These inflammatory processes are closely linked to the production and release of cytokines such as interleukin-1β (IL-1β), tumor necrosis factor-alpha (TNF-α), and monocyte chemoattractant protein-1 (MCP-1) (Frangogiannis 2024; Pan et al. 2024; Wang et al. 2024). The mRNAs of these cytokines can be bound by tristetraprolin (TTP), which promotes their degradation *via* the 3′ untranslated region, thereby reducing mRNA stability, inhibiting translation, and ultimately suppressing cytokine production (Tiedje et al. 2016). Notably, TTP has been identified as a key gene with significantly differential expression between patients with myocardial infarction and healthy controls, suggesting its potentially crucial regulatory role in the inflammatory response to myocardial infarction (Ke et al. 2024). This regulatory role of TTP is closely linked to the activity of mitogen-activated protein kinase-activated protein kinase 2 (MK2), either directly or indirectly, and MK2 deficiency significantly reduces the expression of inflammatory cytokines targeted by TTP  (Ronkina et al., 2010). In addition to its role in regulating TTP, MK2 plays a significant role in myocardial ischemic diseases, with inhibition of MK2 phosphorylation improving left ventricular remodeling and reducing mortality in mice with myocardial infarction (Nakano et al. 2000; Reboll et al. 2017; Pradeep et al. 2024). Given the significant role of the MK2/TTP signaling pathway in myocardial infarction and inflammatory responses, it is proposed that this pathway could serve as a therapeutic target for modulating post-myocardial infarction inflammatory responses.

Qing-Xin-Jie-Yu Granule (QXJYG), a traditional Chinese medicinal formula with a long history of use in China for the treatment of coronary heart disease, is composed of *Astragalus membranaceus* Moench (Fabaceae), *Salvia miltiorrhiza* Bunge (Lamiaceae), *Ligusticum striatum* DC. (Apiaceae), *Agastache rugosa* (Fisch. & C.A. Mey.) Kuntze (Lamiaceae), and *Coptis chinensis* Franch (Ranunculaceae). Previous research has demonstrated that QXJYG can mitigate the inflammatory response and stabilize atherosclerotic plaques in patients with coronary heart disease, alleviating the severity of angina and clinical symptoms such as chest pain and chest tightness (Li 2018; Ju 2019; Cao et al. 2021). In a previous study, the active ingredients in QXJYG were accurately identified using UHPLC-Q-Exactive Orbitrap MS. The analysis revealed that QXJYG contains key compounds such as astragaloside IV, tanshinone IIA, limonin, salvianolic acid B, naringin, and so on (Quan et al. 2023). Among these, the synergistic application of astragaloside IV and tanshinone IIA has been reported to exert potent anti-apoptotic, anti-oxidant, and anti-inflammatory effects by inhibiting the STING signaling pathway, thereby protecting the myocardium from ischemia-reperfusion injury (Zhai et al. 2024). Limonin has been found to reduce infarct size and enhance cardiac function in rats with myocardial infarction, potentially by modulating the expression of genes involved in inflammatory responses, particularly the miRNA rno-miR-10a-5p (Xiong et al. 2021). Additionally, salvianolic acid B alleviates post-ischemic inflammatory responses by inhibiting key proteins in the JNK signaling pathway, thereby reducing damage following myocardial infarction (Lei et al. 2021). Naringin has been demonstrated to mitigate myocardial ischemia-reperfusion injury in rats by decreasing HMGB1 expression and increasing SIRT1 expression, which subsequently reduces the inflammatory response (including IL-23, IL-6, and TNF-α) and oxidative stress (Liu et al. 2022). Collectively, the above evidence supports the therapeutic effect of QXJYG on myocardial infarction, potentially through the suppression of the inflammatory response. However, the precise mechanisms by which QXJYG inhibits the inflammatory response post-myocardial infarction remain incompletely understood. In the present study, we further investigate the anti-inflammatory effects of QXJYG and explore its potential modulation on MK2/TTP signaling pathway using a mouse model of myocardial infarction and H9C2 cardiomyocytes subjected to hypoxic treatment *in vitro*.

## Materials and methods

### Preparation of experimental drug

QXJYG (2112308, Jiangyin Tianjiang Pharmaceutical Co., Ltd., China) is clinically dosed at 22 g/70 kg/day. Isosorbide Mononitrate (ISMN, HY-B0642, MedChemExpress, USA) is clinically dosed at 40 mg/70 kg/day. In accordance with the principles of ‘Pharmacological Experimental Methodology’, the clinical dosages of both drugs were adjusted to appropriate dosages for administration to mice. The drugs were accurately weighed and dissolved in physiological saline to achieve the desired concentrations, which were freshly prepared for gavage administration to the animals. QXJYG and ISMN were dissolved in sterile PBS to create stock solutions at the predetermined concentrations. These stock solutions were aliquoted and stored at −80 °C, and diluted with serum-free DMEM to the working concentrations for cell intervention prior to use.

### Animal experiments

C57BL/6 mice are commonly used in left anterior descending coronary artery (LAD) ligation models due to their stable infarct size, well-characterized physiological responses, and reliable post-infarction remodeling. This strain exhibits cardiac remodeling and inflammatory responses similar to humans, making it suitable for studying ischemic heart disease and potential treatments. In this study, male C57BL/6 mice (10–12 weeks old, 20–22 g) were purchased from Shanghai Slack Experimental Animals Co., Ltd (Approval No.: SCXK (Hu) 2022-0004). The mice were housed under standard conditions, with a controlled room temperature of 24 ± 2 °C, humidity of 50–60%, with a 12 h light/dark cycle. The animals were acclimatized for 5 days prior to the start of the study. A total of 48 C57BL/6 mice were randomly assigned to the following groups based on body weight:① Sham (received saline, ig), ② Model (myocardial infarction, received saline, ig),③ Model + ISMN (ISMN, 5.2 mg/kg/day, ig), ④ Model + QXJYG-L (QXJYG-L, 1.43 mg/g/day, low dose, ig), ⑤ Model + QXJYG-M (QXJYG-M, 2.86 mg/g/day, medium dose, ig), and ⑥ Model + QXJYG-H (QXJYG-H, 5.72 mg/g/day, high dose, ig), with 8 animals in each group. Mice subjected to LAD ligation were performed to establish the model of myocardial infarction following the methodology previously described by Shen et al. (2020). Briefly, mice with pre-plucked chest hair were anaesthetised with 1% isoflurane gas at a flow rate of 0.6–1 litres/min, and then ligated 2–3 mm below the left auricle with a sterile, non-absorbable, banded suture needle (size 6–0). Successful ligation was confirmed by observing tissue discoloration in the apical region below the ligated area. The surgical procedures for the Sham group mirrored those of the Model group, except that no ligation was performed. After surgery, the animals were kept on a 30 °C heating pad until fully recovered, then returned to their housing cages. The experimental protocols involving mice were conducted in compliance with the ARRIVE (Animal Research: Reporting of *In Vivo* Experiments) guidelines to ensure transparent and accurate reporting. This study is part of a larger overarching project that has received ethical approval, allowing for multiple related publications under a single Ethics Approval. The study was reviewed and approved by the Animal Ethics Committee of the Research Institute at Fujian University of Traditional Chinese Medicine (FJTCM IACUC 2022197) on October 24, 2022. All procedures were performed in accordance with relevant ethical standards and regulations.

### In vitro culture of the H9C2 myocardial cell line

H9C2 cells (Rattus norvegicus, embryonic heart, CVCL 0286, SCSP-5211), purchased from Wuhan Procell Life Science and Technology Co., Ltd., were cultured in DMEM supplemented with 10% FBS and 1% Penicillin-Streptomycin (100×) at 37 °C, in a 5% CO_2_ atmosphere with saturated humidity until reaching 80–90% confluence for passaging and seeding. For experiments, cells were first seeded into 10^3^ cells/well in 96-well plates to determine optimal drug concentrations using CCK-8 assay. Subsequently, they were seeded at 8 × 10^4^ cells/well in 6-well plates for western blot or qPCR experiments, grouped as follows: ①Control (normoxia); ②Model (hypoxia); ③ISMN (hypoxia + 900 μM ISMN); ④ QXJYG-L (hypoxia + 7.5 μg/mL QXJYG); ⑤ QXJYG-M (hypoxia + 15 μg/mL QXJYG); ⑥QXJYG-H (hypoxia + 30 μg/mL QXJYG). After a 24 h period of plating, cells were treated with the respective medications and exposed to 24 h of hypoxia. The hypoxic conditions were established based on previous laboratory data: 1%O_2_, 94% N_2_ and 5% CO_2_. Following the completion of hypoxic culture, both the cells and their corresponding culture medium were harvested for further experimental procedures.

### Cardiac echocardiography

After a 4-week medication regimen, the cardiac function of mice was assessed using the high-resolution Vevo2100 ultrasound system. Mice were anesthetized with isoflurane after chest hair removal and positioned supine on an animal operating platform. The MS400 ultrasound probe was placed on the left side of the mouse’s sternum at a 45° angle to the midline to obtain a long-axis view of the left ventricle. The platform was then tilted with the top left corner lower than the bottom right to align the probe along the direction of the mouse’s cardiac apex, which allowed for capturing images of the four cardiac chambers. A pulsed-wave (PW) Doppler sample volume was positioned at the site of the highest blood flow velocity to record mitral valve hemodynamics. Cardiac function was analyzed using Vevo LAB offline software, with measurements averaged over three cardiac cycles to determine parameters including ejection fraction (EF), fractional shortening (FS), left ventricular internal diameter in systole (LVIDs), left ventricular internal diameter in diastole (LVIDd), and myocardial performance index (MPI).

### Sample collection

Upon completion of the echocardiogram examination, the mice were anesthetized with isoflurane, and blood was collected from the orbital venous plexus. Following blood collection, the mice were euthanized using an overdose of isoflurane. The hearts were then excised and sectioned along the longitudinal plane into two halves: one containing the apical region and the other containing the basal region. The apical portion was rinsed with saline, then fixed in a 4% paraformaldehyde solution for 48 h, and subsequently embedded in paraffin for histological examination. The basal portion of the heart was stored at −80 °C for western blot analysis. Blood samples were centrifuged (4 °C, 3000 rpm, 15 min) to isolate the serum, which was then stored at −80 °C for subsequent experiments.

### Histopathological analysis

Paraffin-embedded sections (4 μm thick) were stained with hematoxylin and eosin (HE) (Solarbio, Beijing, China) to visualize pathological changes in myocardial tissue. Additionally, Masson’s trichrome staining (Solarbio, Beijing, China) was performed to assess the collagen volume fraction (CVF). The stained sections were examined using an optical microscope (Leica, Germany) at 400 × magnification. The CVF was quantified using microscopic image analysis software (Image J, National Institutes of Health, Bethesda, Maryland, USA).

### Enzyme‑linked immunosorbent assay

Serum samples were analyzed using enzyme-linked immunosorbent assay (ELISA) to quantify cardiac biomarkers, including creatine kinase isoenzyme MB (CK-MB, MM-43703M1), cardiac troponin T (cTnT, MM-44145M1), and brain natriuretic peptide (BNP, MM-0060M1). These ELISA kits were procured from Jiangsu Enzyme Immuno Industrial Co., Ltd., China. The ELISA detection procedure was performed in strict accordance with the guidelines provided in the respective ELISA kit instructions. Briefly, samples and standards were added to pre-coated plates, followed by incubation with specific detection antibodies. After washing, a substrate solution was added, and the reaction was stopped upon color development. Absorbance was measured at the appropriate wavelength using a microplate reader.

### Bio-Plex Pro Mouse Chemokine assay

Cytokine concentrations in both mouse serum and cardiac tissue lysate supernatants were measured using the Bio-Plex Pro Mouse Chemokine assay. The assay was conducted in strict accordance with the Bio-Plex Pro Mouse Chemokine assays Kit instructions (M60009RDPD; Bio-Rad, Hercules, CA, USA). The Bio-Plex Pro Mouse Chemokine Assays were conducted using the Bio-Plex 200 system (Bio-Rad, USA). Samples, standards, and detection beads were prepared according to the manufacturer’s instructions. Following incubation and washing steps, the beads were resuspended in assay buffer, and data acquisition were performed. The data obtained from the assay were then meticulously exported for further analysis using Bio-Plex Manager software.

### Cell counting Kit-8 assay

After seeding H9C2 cells into a 96-well plate and incubating for 24 h, the original culture medium was replaced. The cells were then grouped and treated with varying concentrations of QXJYG or ISMN prepared in DMEM and incubated for an additional 24 h. Following the incubation period, 10 μL of CCK-8 reagent (KTC011001; Abbkine, USA) was added to each well, and the plates were further incubated for 1 h. Finally, the optical density of each group was measured using a microplate reader to assess cell viability and determine the optimal intervention concentration of the drugs.

### Western blot analysis

Minced heart tissue or H9C2 cells were collected and lysed using Western & IP Lysis Buffer (P0013, Beyotime, China) supplemented with protease inhibitors (Cocktail, HY-K0010, MedChemExpress, USA), phosphatase inhibitors (PhosSTOP, 04906845001, Roche Diagnostics GmbH, Germany), and a serine protease inhibitor (PMSF, P0100-10, Beijing Solarbio Life Science Co., Ltd., China). The mixture was lysed on ice for 30 min and then centrifuged at 12,000 × g for 15 min at 4 °C to obtain the supernatant. Total protein (40 µg) was separated on a 10% SDS-polyacrylamide gel and then transferred onto a 0.45 µm PVDF membrane (10600029, GE Healthcare, Germany). After blocking with a rapid protein-free solution (PS108P, Epizyme Biotech Co., Ltd.) for 30 min, the membrane was then incubated overnight at 4 °C with primary antibodies [rabbit anti-p-MK2 (1:1000 dilution, AF7309, AB_2843749, Affinity Biosciences, USA), rabbit anti-MK2 antibody (1:1000 dilution, #3042, AB_10694238, Cell Signaling Technology, USA), mouse anti-TTP (1:1000 dilution, MA5-25406, AB_ 2725702 Thermo Fisher Scientific, USA), and rabbit anti-Vinculin (1:1000 dilution, GTX113294, AB_10732545, GeneTex, USA)]. Following TBST washes, secondary antibodies [rabbit secondary antibody (L3012, AB_895483, SAB, USA) and mouse secondary antibody (L35665, AB_ 3665961, SAB, USA)] were applied for 1 h at room temperature. Protein bands were visualized using an ECL detection kit (Thermo Fisher Scientific, USA) and quantified using ImageJ software.

### RT-qPCR analysis

Total RNA was isolated from H9C2 cells using the Total RNA Extraction Kit (DP451, Tiangen Biochemical Technology Co., Ltd., China) and reverse-transcribed into cDNA using the One-Step Reverse Transcription Kit (KR118, Tiangen Biochemical Technology Co., Ltd., China). cDNA samples were then amplified using the Fluorescent Quantitative Detection Kit (FP205, Tiangen Biochemical Technology Co., Ltd., China) with gene-specific primers as listed in Table 1. Quantitative PCR was performed on the CFX96^™^ Touch Real-Time PCR Detection System (Bio-Rad Laboratories, Inc.). Gene expression levels were normalized to β-actin, and relative mRNA levels were calculated using the 2^−ΔΔCT^ method.

**Table 1.** primer sequences.

Primer name	Sequence (5′–3′)	Lth (bp)
TNF-α-F	CCTCACCCACACCGTCAG	170
TNF-α-R	GCAGGTCCCCCTTCTCCA	
IL-6-F	GACTTCCAGCCAGTTGCCTT	112
IL-6-R	CTGGTCTGTTGTGGGTGGTAT	
IL-1β-F	GCTACCTATGTCTTGCCCGT	123
IL-1β-R	TCACACACTAGCAGGTCGTC	
β-actin-F	CGCGAGTACAACCTTCTTGC	211
β-actin-R	CCTTCTGACCCATACCCACC	

### Statistical analysis

All data were analyzed using SPSS 23.0. Metric data conforming to a normal distribution were represented as the $\bar{x}\pm s$,while non-normally distributed data were expressed as the median and interquartile range. The Shapiro–Wilk test was initially used to assess the normality of the data. For normally distributed data, a one-way analysis of variance (ANOVA) was conducted to compare multiple groups. Post-hoc analysis was performed using the Least Significant Difference (LSD) test for homogeneous variances, and the Games-Howell test was for unequal variances. For non-normally distributed data, the Kruskal–Wallis One-Way ANOVA was employed. The Bonferroni correction was applied for multiple comparisons. And the results were considered statistically significant when *P* values were less than 0.05.

## Results

### QXJYG improves cardiac function in mice with myocardial infarction

**Figure 1.**
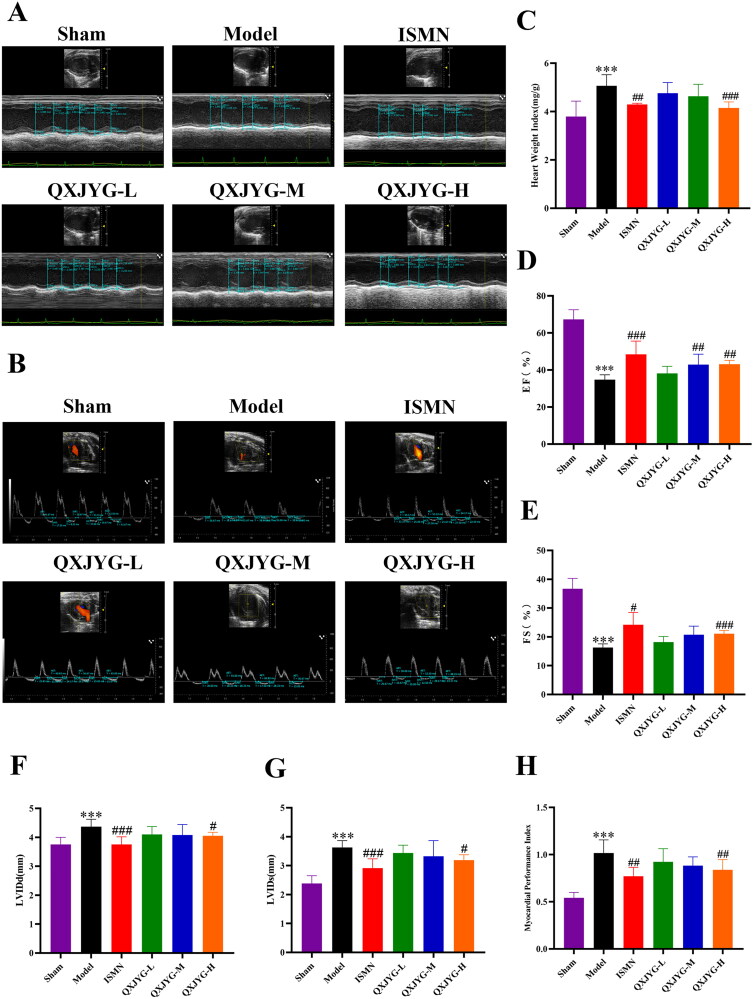
QXJYG improves cardiac function in mice after myocardial infarction surgery. (A and B) Representative images of echocardiographic in each group were presented. (C) The heart weight index (heart weight/body weight) was calculated and presented as a histogram (*n* = 6). (D–G) The echocardiography measurement of EF, FS, LVIDd, LVIDs was shown (*n* = 6). (H) The echocardiography measurement of MPI was shown (*n* = 5). ***P < 0.001,vs. Sham group. ^#^*P* < 0.05; ^##^*P* < 0.01; ^###^*P* < 0.001,vs. Model group. EF: ejection fraction; FS: fractional shortening; LVIDd: ventricular internal diameter in diastole; LVIDs: left ventricular internal diameter in systole; MPI: myocardial performance index.

Four weeks following myocardial infarction surgery, the heart weight index (HWI) was significantly increased in the Model group compared to the Sham group (Figure 1(C); ***P < 0.001, vs. Sham group). Echocardiographic analysis revealed that both the EF and FS were markedly reduced in the Model group, while the LVIDs, LVIDd, and MPI were significantly increased (Figure 1(D–F); ***P < 0.001, vs. Sham group). These findings indicated the successful establishment of the myocardial infarction model. ISMN treatment mitigated the reductions in EF and FS and decreased HWI, LVIDs, LVIDd, and MPI (Figure 1(C–H); ^#^*P <* 0.05; ^##^*P <* 0.01; ^###^*P <* 0.001, vs. Model group). Treatment with both medium and high doses of QXJYG significantly increased EF (Figure 1(D); ^##^*P <* 0.01, vs. Model group). Additionally, high doses of QXJYG led to significant increases in FS and notably reduced HWI, LVIDs, LVIDd, and MPI (Figure 1(C–H); ^#^*P* < 0.05; ^##^*P* < 0.01; ^###^*P* < 0.001, vs. Model group). These results demonstrate that QXJYG treatment significantly ameliorates myocardial infarction-induced cardiac dysfunction in mice, with effects comparable to those of ISMN.

### QXJYG improves pathological changes and myocardial injury markers in mice with myocardial infarction

**Figure 2.**
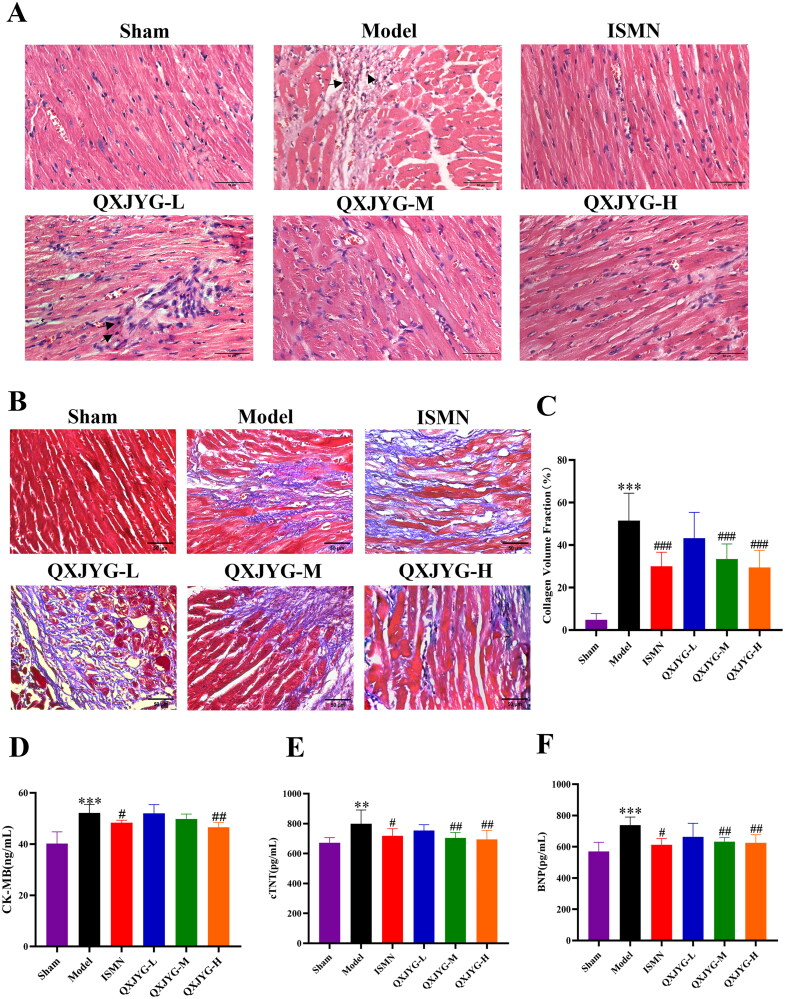
QXJYG improves pathological changes of heart and the markers of myocardial injury in mice after myocardial infarction surgery. (A) Representative images of HE staining of each group (scale bar = 50 μm) were displayed; The area indicated by the black arrow showed inflammatory cell infiltration; Magnification, ×400. (B and C) Masson’s trichrome–stained histological pictures and quantitation of the area of fibrosis (blue) in cardiac tissues of mice from each group (scale bar = 50 μm) were displayed. Magnification, ×400. (D–F) CK-MB, cTnT and BNP levels in the serum of each group were determined by ELISA kits, respectively (*n* = 5). **P < 0.01;***P < 0.001,vs. Sham group. ^#^*P* < 0.05; ^##^*P* < 0.01; ^###^*P* < 0.001,vs. Model group. CK-MB: creatine kinase isoenzyme MB; cTnT: cardiac troponin T; BNP: brain natriuretic peptide.

The HE staining results showed that in the Sham group, the myocardial cells were neatly arranged in a spindle-shaped pattern, with centrally located oval nuclei. The cytoplasm was stained red, and the nuclei were stained purple-blue. In contrast, the Model group exhibited thickened myocardial fibers, disordered arrangement, enlarged interstitial spaces, and pathological changes such as vacuolization and infiltration of interstitial inflammatory cells (as indicated by arrows); these pathological changes were ameliorated following intervention with low, medium, and high doses of QXJYG and ISMN (Figure 2(A)). Masson’s trichrome staining revealed that in the Sham group, the myocardial cells were orderly arranged with minimal collagen fiber deposition, which did not disrupt myocardial structure. However, in the Model group, substantial collagen fiber deposition, irregular myocardial cell arrangement, and structural disarray were observed (Figure 2(B–C); ***P < 0.001, vs. Sham group). After treatment with ISMN, as well as medium and high dose of QXJYG, collagen fiber deposition was reduced, and myocardial structure improved (Figure 2(B–C), ^###^*P <* 0.001, vs. Model group). These results indicate that treatment with QXJYG alleviates pathological damage and improves collagen fiber deposition in post-myocardial infarction mice, with effects comparable to ISMN.

Biochemical analysis showed that compared to the Sham group, the serum levels of CK-MB, cTnT, and BNP had significantly elevated in Model group (Figure 2(D–F), **P < 0.01, ***P < 0.001 vs. Sham group). Compared to the Model group, treatment with ISMN and high-dose QXJYG significantly reduced serum CK-MB levels (Figure 2(D), ^#^*P* < 0.05; ^##^*P <* 0.01, vs. Model group). Additionally, the ISMN group, along with the medium and high doses of QXJYG, significantly decreased serum cTnT levels (Figure 2(E), ^#^*P* < 0.05; ^##^*P <* 0.01, vs. Model group). Furthermore, the ISMN group, as well as the medium and high dose of QXJYG groups, showed a statistically significant decrease in serum levels of BNP (Figure 2(F), ^#^*P* < 0.05; ^##^*P* < 0.01, vs. Model group). These findings indicate that QXJYG can reduce the levels of myocardial injury-specific markers BNP, cTnT, and CK-MB in the serum of mice with myocardial infarction, with efficacy comparable to ISMN.

### QXJYG alleviates inflammatory cytokine levels in the hearts and serum of myocardial infarction-induced mice

**Figure 3.**
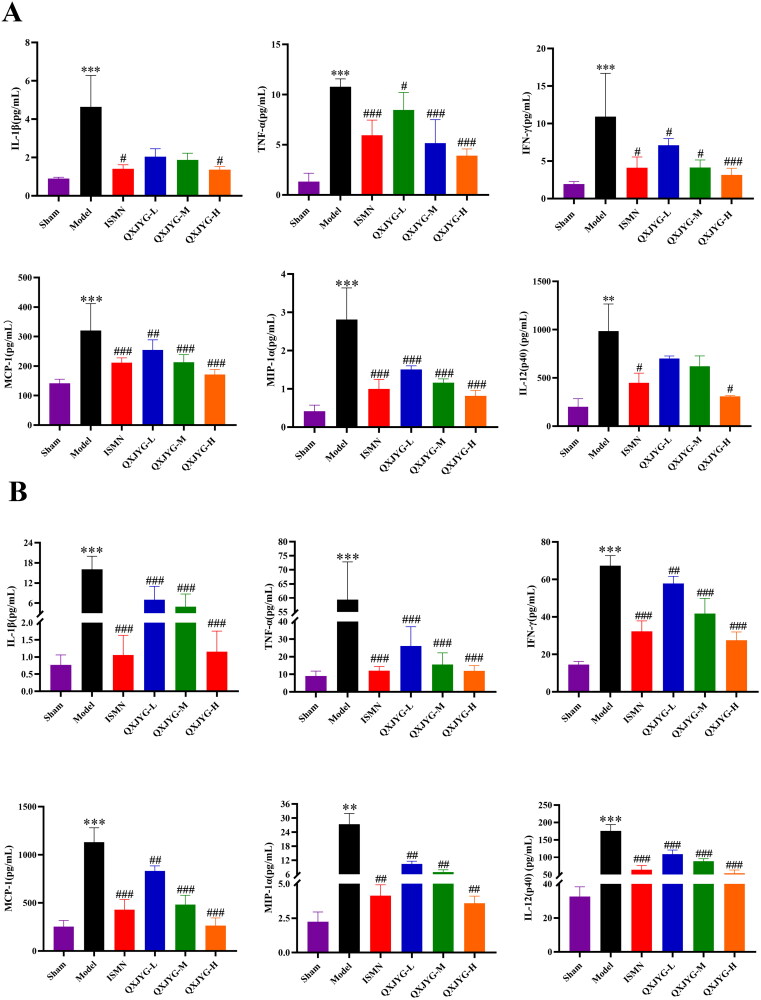
QXJYG alleviates inflammatory cytokine levels both in the serum and cardiac tissue of mice after myocardial infarction surgery. (A and B) Levels of pro-inflammatory factors IL-1β, TNF-α, IFN-γ, MCP-1, MIP-1α, and IL-12(p40) in the serum and cardiac tissue of each group were performed via Bio-Plex pro mouse chemokine assay, respectively. **P < 0.01; ***P < 0.001,vs. Sham group. ^#^*P* < 0.05; ^##^*P* < 0.01; ^###^*P* < 0.001,vs. Model group. IL: interleukin; TNF: tumor necrosis factor; IFN: interferon; MCP: monocyte chemoattractant protein; MIP: macrophage inflammatory protein.

The Bio-Plex Pro Mouse Chemokine assay results showed that compared to the Sham group, the serum levels of IL-1β, TNF-α, interferon-γ (IFN-γ), MCP-1, interleukin-12 (p40) (IL-12(p40)), and macrophage inflammatory protein-1α (MIP-1α) in the Model group were significantly elevated (Figure 3(A), **P < 0.01; ***P < 0.001, vs. Sham group). Compared to the Model group, the ISMN group significantly reduced the levels of these cytokines in serum (Figure 3(A), ^#^*P* < 0.05; ^###^*P* < 0.001, vs. Model group). Additionally, low, medium, and high doses of QXJYG significantly decreased the serum levels of TNF-α, IFN-γ, MCP-1, and MIP-1α, with only the high dose of QXJYG able to reduce the serum levels of IL-1β and IL-12(p40) (Figure 3(A), ^#^*P* < 0.05; ^##^*P* < 0.01; ^###^*P* < 0.001, vs. Model group).

In cardiac tissue, compared to the Sham group, the levels of IL-1β, TNF-α, IFN-γ, MCP-1, IL-12(p40), and MIP-1α were significantly elevated in the Model group mice (Figure 3(B), **P < 0.01; ***P < 0.001, vs. Sham group). Compared to the Model group, the ISMN and all doses of QXJYG significantly reduced these inflammatory cytokine levels in cardiac tissue (Figure 3(B), ^##^*P* < 0.01; ^###^*P* < 0.001, vs. Model group).

These findings indicate that QXJYG intervention can reduce the levels of multiple inflammatory cytokines both in the serum and cardiac tissue of myocardial infarction-induced mice, highlighting its significance in alleviating post-myocardial infarction inflammatory responses.

### QXJYG regulates the MK2/TTP signaling pathway in the hearts of myocardial infarction-induced mice

**Figure 4.**
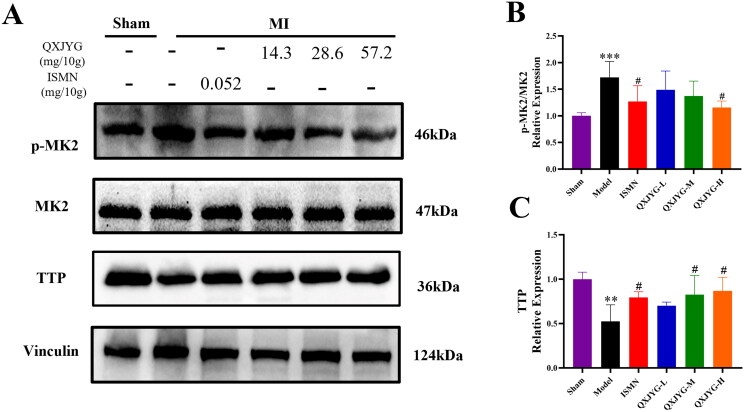
QXJYG regulates the MK2/TTP sgnaling pathway in the cardiac tissues of mice after myocardial infarction surgery. (A-C) The protein levels of p-MK2, MK2, TTP and Vinculin were measured via western blot analysis (*n* = 3); Vinculin was used as the internal control. **P < 0.01; ***P < 0.001,vs. Sham group. ^#^*P* < 0.05,vs. Model group. MK2: Mitogen-activated protein kinase-activated protein kinase 2; TTP: tristetraprolin.

Western Blot analysis revealed that, compared to the Sham group, the ratio of p-MK2/MK2 in the cardiac tissue of the Model group was significantly increased (Figure 4(A–B), ***P < 0.001, vs. Sham group), while the protein expression of TTP was significantly decreased (Figure 4(A and C), **P < 0.01, vs. Sham group). The ratio of p-MK2/MK2 was significantly decreased in the ISMN and high dose of QXJYG groups compared to the Model group (Figure 4(A–B), ^#^*P* < 0.05, vs. Model group). Additionally, the protein expression of TTP was significantly increased in the ISMN, medium, and high dose of QXJYG groups (Figure 4(A and C), ^#^*P <* 0.05, vs. Model group). These findings suggest that QXJYG can significantly modulate the MK2/TTP signaling pathway, potentially influencing the inflammatory response following myocardial infarction.

### QXJYG reduces inflammatory cytokine levels in hypoxia-induced H9C2 cells via the modulation of MK2/TTP signaling pathway

**Figure 5.**
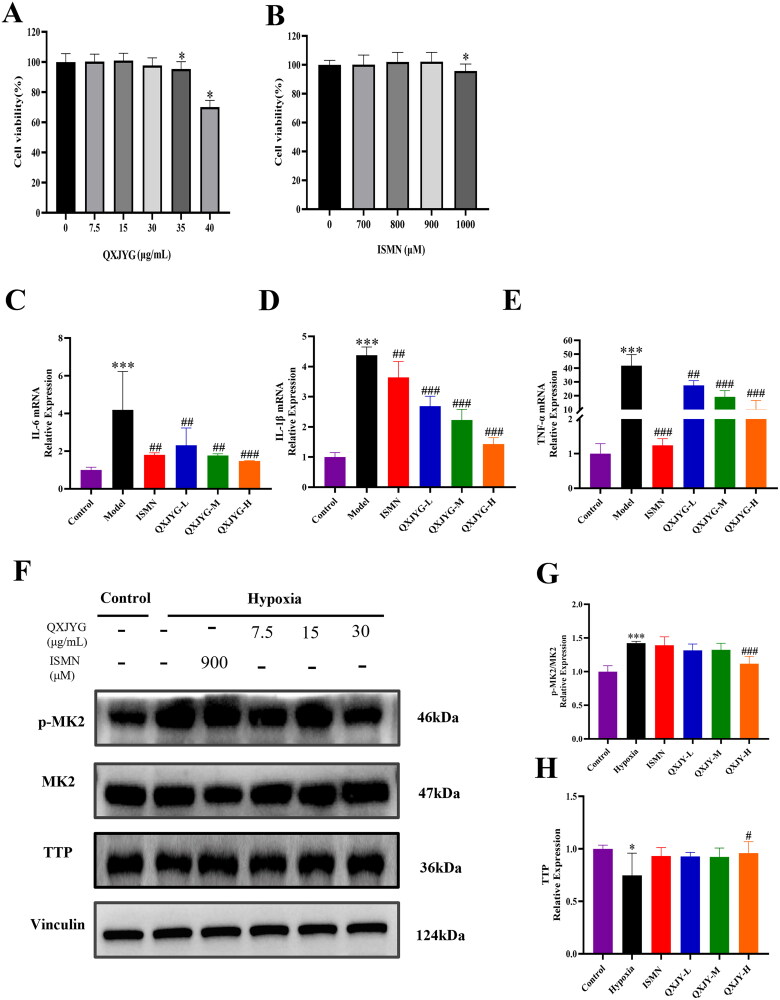
QXJYG reduces inflammatory cytokine content in hypoxia-induced H9C2 cells via the MK2/TTP pathway suppression. (A and B) The optimal concentrations of QXJYG and ISMN intervention in H9C2 cells was evaluated by CCK-8 assay, respectively. (C–E) The mRNA levels of pro-inflammatory factors IL-6, IL-1β and TNF-α were measured via RT-qPCR analysis (*n* = 3). (F–H) The protein levels of p-MK2, MK2, TTP and Vinculin were measured via western blot analysis (*n *= 3); Vinculin was used as the internal control. *P < 0.05; ***P < 0.001,vs. Control group. ^#^ *P*< 0.05; ^##^*P* < 0.01; ^###^*P* < 0.001,vs. Model group. IL: interleukin; TNF: tumor necrosis factor; MK2: mitogen-activated protein kinase-activated protein kinase 2; TTP: tristetraprolin.

To further illustrate the efficacy of QXJYG and the mechanism of action of its anti-inflammation, an *in vitro* hypoxia-induced H9C2 cell model was established. Firstly, the optimal concentrations of QXJYG and ISMN for intervention in H9C2 cells were determined using the CCK-8 assay. As shown in Figure 5(A–B), no significant differences in cell viability were observed for QXJYG at concentrations of 0–30 μg/mL and for ISMN at concentrations up to 900 μM compared to the Control group, indicating no cytotoxicity at these levels. Therefore, the selected low, medium and high concentrations of QXJYG for further experiments were 7.5, 15 and 30 μg/mL, respectively, while the concentration of ISMN was 900 μM.

Secondly, RT-qPCR analysis was performed to evaluate the anti-inflammatory effect of QXJYG in hypoxia-induced H9C2 cells. Compared to the Control group, the Model group exhibited significantly elevated mRNA levels of IL-1β, IL-6, and TNF-α after 24 h of hypoxia induction (Figure 5(C–E), ***P < 0.001, vs. Control group). Whereas, ISMN and QXJYG treatment resulted in a significant reduction in the mRNA levels of IL-1β, IL-6, and TNF-α (Figure 5(C–E), ^##^*P* < 0.01; ^###^*P* < 0.001, vs. Model group). These results indicate that QXJYG effectively mitigates inflammatory response in the hypoxia-induced H9C2 cells.

Finally, western blot analysis was further conducted to assess whether QXJYG could inhibit hypoxia-induced inflammation by regulating the MK2/TTP signaling pathway. The results indicated that the Model group exhibited a significant increase in the ratio of p-MK2/MK2 and a significant decrease in TTP protein levels, compared to the Control group (Figure 5(F and H), *P < 0.05; ***P < 0.001, vs. Control group). However, the high dose of QXJYG group demonstrated a statistically significant reduction in the p-MK2/MK2 ratio and an increase in TTP protein expression compared to the Model group (Figure 5(F–H), ^#^*P* < 0.05; ^###^*P* < 0.001, vs. Model group). Taken together, these results suggest that QXJYG may attenuate the inflammatory response following hypoxia-induced myocardial injury by modulating the MK2/TTP signaling pathway.

## Discussion

Myocardial infarction is caused by the heart’s limited regenerative capacity, leading to irreversible cardiomyocyte death, which current treatments are insufficient to reverse. Despite significant advancements in stem cell therapy and engineered extracellular vesicle therapy, the clinical challenges posed by myocardial no-reflow and reperfusion injury remain unresolved, contributing to persistently high mortality rates during acute myocardial infarction hospitalization (Lee et al. 1993; Solomon et al. 2005). Against this backdrop, integrating traditional Chinese medicine principles alongside modern medical advancements, the research team led by Academician Chen Keji closely correlated elevated inflammatory markers in coronary heart disease with the toxic pathogenesis theory, proposing the hypothesis of ‘blood stasis and toxin causing catastrophe’(Xu et al. 2008). They innovatively developed a theoretical framework for the etiology and pathogenesis of coronary heart disease, leading to the proposal of QXJYG, primarily characterized by its ‘activating blood circulation and detoxifying’ effects and widely used in China for treating cardiovascular disease, demonstrating favorable clinical outcomes. Clinical studies have demonstrated that the combination of QXJYG with conventional western medicine can effectively inhibit inflammatory cytokine levels, improve hemorheological properties and cardiac function, and protect vascular endothelial function in patients with coronary heart disease, thereby reducing the risk of cardiovascular events (Gao 2017; Li 2018; Li et al. 2019). Both *in vitro* and *in vivo* experiments have shown that QXJYG inhibits NLRP3 inflammasome activation, modulates macrophage pyroptosis, and reduces the release of IL-1β and IL-18 in atherosclerotic mouse models, while also regulating lipid metabolism to stabilize vulnerable atherosclerotic plaques (Ju 2019; Wang 2019). Additionally, QXJYG has been shown to improve cardiac remodeling following myocardial infarction, potentially through the regulation of mitochondrial autophagy (Jiang 2022). However, the precise mechanism by which QXJYG reduces post-myocardial infarction inflammation remains unclear. Our study indicates that QXJYG treatment significantly enhances cardiac function, alleviates cardiac pathological changes, and suppresses inflammatory factors levels in a myocardial infarction-induced mouse model, potentially through modulation of the MK2/TTP signaling pathway.

Following myocardial infarction, an immediate inflammatory response is triggered at the infarct site, characterized by the rapid accumulation of immune cells such as neutrophils, monocytes, and macrophages, which release various cytokines and chemokines, including IL-1β, MCP-1, MIP-1α, IFN-γ, IL-12, and TNF-α Wang et al. 2018). These pro-inflammatory mediators are initially released locally at the site of tissue injury and subsequently enter the surrounding vasculature, leading to their systemic dissemination through the circulatory system (Dewald et al. 2005; Chang et al. 2024). While a moderate inflammatory response can limit infarct size and promote recovery of ischemic myocardium, excessive inflammatory reactions can lead to substantial degradation of matrix components and fibrotic scar formation, ultimately hindering myocardial regeneration and cardiac repair (Chang et al. 2024; Wang et al. 2018). In line with these findings, our study demonstrates that QXJYG reduces the levels of inflammatory cytokines in both the heart and serum of mice with myocardial infarction , improving cardiac function, and mitigating myocardial fibrosis in the model.

RNA-binding proteins (RBPs) are key regulators of RNA metabolism, including maturation, transport, localization, and translation. Recent research increasingly highlights their role in anti-inflammatory effects by targeting and degrading mRNA transcripts of inflammatory mediators, thereby reducing the expression of inflammatory factors (Rezcallah et al. 2021). For instance, RBPs such as TTP facilitate mRNA degradation, thereby limiting the production of pro-inflammatory factors (Sandler et al. 2011). It has been previously reported that TTP knockout mice display a more pronounced inflammatory response compared with their wild-type littermates (Taylor et al. 1996). Moreover, suppression the TTP expression has been associated with worsened heart failure prognosis due to elevated levels of inflammatory factors, such as TNF-α and IL-6 (Streicher et al. 2010). This study aimed to evaluate whether QXJYG could modulate the expression of inflammatory factors and TTP in a myocardial infarction model. The results revealed that TTP expression was decreased, while inflammatory factors levels were elevated, in both myocardial infarction model mice and hypoxia-induced H9C2 cells compared with control groups. However, QXJYG treatment upregulated TTP expression and decrease inflammatory factors levels. These findings suggest that TTP is involved in the inflammatory response following myocardial infarction, and QXJYG may exert anti-inflammatory effects by upregulating the RNA-binding protein TTP.

During acute myocardial ischemic injury, cardiomyocytes experience alterations in intracellular and extracellular osmolarity, dysregulation of ion homeostasis, depletion of energy metabolites including ATP and phosphocreatine, and activation of inflammatory cytokines (Frangogiannis 2012; Kologrivova et al. 2021; Chang et al. 2023). These factors can trigger kinase signaling cascades, ultimately culminating in the phosphorylation and activation of MK2. Previous studies have demonstrated that MK2 deletion or inhibition diminishes the production of inflammatory cytokines, indicating that MK2 plays a crucial regulatory role in the inflammatory response (Mahtani et al. 2001; Hitti et al. 2006). Inhibition of MK2 phosphorylation has also been shown to attenuate cardiac hypertrophy and heart failure, improve left ventricular remodeling, and decrease mortality in post-myocardial infarction mouse models (Cheng et al. 2021). Moreover, the MK2/p-MK2 cascade regulates the stability of ARE-containing mRNAs, partly because MK2 is a key regulator of the expression, stability, and function of TTP (Tiedje et al. 2012; Prabhala and Ammit 2015). Activated MK2 phosphorylates critical serine residues in TTP (Ser52 and Ser178 in mice, Ser186 in humans), promoting TTP ubiquitination and subsequent degradation *via* the lysosomal pathway, which ultimately leads to its inactivation (Bak and Mikkelsen 2010). Furthermore, phosphorylated TTP binds to 14-3-3 proteins, an interaction that hinders TTP’s normal association with target mRNAs, thereby disrupting the TTP-mediated mRNA degradation process (Stoecklin et al. 2004). Consequently, MK2-mediated phosphorylation of TTP inhibits its function, preventing the degradation of mRNA of pro-inflammatory factors. Studies have shown that MK2 inhibitors, such as CC-99677, inhibit MK2 activity and thus prevent TTP phosphorylation, maintaining TTP in its active state, allowing it to continue recognizing and degrading mRNAs of inflammatory cytokines, such as TNF, IL-6, and IL-1β (Gaur et al. 2022). Therefore, modulation of the MK2/TTP signaling pathway can effectively regulate inflammatory responses by inhibiting TTP phosphorylation and preserving its ability to degrade inflammation-related mRNAs. (Wu et al. 2013). Our study demonstrates that QXJYG reduces MK2 phosphorylation and activation in cardiomyocytes induced by myocardial infarction and hypoxia, while simultaneously increasing TTP levels. These findings suggest that the anti-inflammatory effects of QXJYG are mediated through the modulation of the MK2/TTP signaling pathway.

An increasing body of experimental and clinical evidence suggests that anti-inflammatory therapies targeting cytokines and chemokines not only reduce the risk of myocardial infarction in patients with existing cardiovascular diseases or those at high cardiovascular risk but also improve the prognosis of patients who have already experienced a myocardial infarction (Li et al. 2023; Matter et al. 2024). Building on the previous findings that QXJYG improves the prognosis in a myocardial infarction mouse model, our study further demonstrates that the suppression of cardiac inflammation may be a key mechanism underlying this protective effect (Chen et al. 2024). Through *in vivo* and *in vitro* experiments, we further elucidated that the beneficial effects of QXJYG on reducing post-myocardial infarction and hypoxia-induced inflammation may be mediated *via* the regulation of the MK2/TTP signaling pathway. These findings establish a robust scientific foundation for the clinical application of QXJYG in the treatment of myocardial infarction, unveiling a novel anti-inflammatory approach that holds promise for application in other inflammation-related cardiovascular diseases. Furthermore, they serve as a pivotal benchmark for analogous research endeavors.

Nonetheless, this study is accompanied by several limitations. Firstly, our exploration of QXJYG’s effects on myocardial infarction and hypoxia-induced cardiomyocyte models, the utilization of a single animal model and cell line restricts the generalizability of our findings. Incorporating different animal models, such as ischemia/reperfusion injury models, and other cardiomyocyte or heart cell lines would be essential to validate the broader applicability of the results. Secondly, while the study underscores the potential involvement of the MK2/TTP signaling pathway, the detailed molecular mechanisms underlying this regulatory effect remain to be fully understood. Subsequent research endeavors could incorporate gene editing technologies to systematically knock out or down key components of the MK2/TTP axis in myocardial infarction mice. Thirdly, considering the multi-target and multi-pathway characteristics of traditional Chinese medicine, future studies ought to delve into mechanisms beyond inflammation, offering deeper insights into myocardial infarction treatment and broadening the therapeutic horizon of QXJYG. This approach would allow for a more precise elucidation of the specific molecular targets through which QXJYG exerts its regulatory effects, thereby facilitating more targeted medical interventions.

## Conclusions

In conclusion, our study demonstrated QXJYG effectively improved cardiac function, alleviated cardiac pathological injury, fibrosis and inflammatory response after myocardial infarction surgery. The modulation of the MK2/TTP signaling pathway appeared to be a key mechanism underlying the beneficial effects of QXJYG against myocardial infarction.

## Supplementary Material

supplementary material.docx

## Data Availability

The datasets generated and analyzed during the current study are available from the corresponding author upon reasonable request. Additionally, any supplementary materials referenced in the manuscript are provided as supplementary files.

## References

[CIT0001] Bak RO, Mikkelsen JG. 2010. Regulation of cytokines by small RNAs during skin inflammation. J Biomed Sci. 17(1):53. doi: 10.1186/1423-0127-17-53. 20594301 PMC2905360

[CIT0002] Cao C, Wang R, Gao D, Zhou Y, Lv Y, Ma L. 2021. Therapeutic efficacy of Gui Feng Tong Yu Tang combined with western drugs in the treatment of acute myocardial infarction and its effect on patients’ blood rheological indexes and cardiac function. Shanxi J Tradit Chin Med. 42(10):1382–1384. Chinese.

[CIT0003] Chang X, Li Y, Liu J, Wang Y, Guan X, Wu Q, Zhou Y, Zhang X, Chen Y, Huang Y, et al. 2023. β-tubulin contributes to Tongyang Huoxue decoction-induced protection against hypoxia/reoxygenation-induced injury of sinoatrial node cells through SIRT1-mediated regulation of mitochondrial quality surveillance. Phytomedicine. 108:154502. doi: 10.1016/j.phymed.2022.154502. 36274412

[CIT0004] Chang X, Liu R, Li R, Peng Y, Zhu P, Zhou H. 2023. Molecular mechanisms of mitochondrial quality control in ischemic cardiomyopathy. Int J Biol Sci. 19(2):426–448. doi: 10.7150/ijbs.76223. 36632466 PMC9830521

[CIT0005] Chang X, Zhang Q, Huang Y, Liu J, Wang Y, Guan X, Wu Q, Liu Z, Liu R. 2024. Quercetin inhibits necroptosis in cardiomyocytes after ischemia-reperfusion via DNA-PKcs-SIRT5-orchestrated mitochondrial quality control. Phytother Res. 38(5):2496–2517. doi: 10.1002/ptr.8177. 38447978

[CIT0006] Chang X, Zhou S, Liu J, Wang Y, Guan X, Wu Q, Liu Z, Liu R. 2024. Zishenhuoxue decoction-induced myocardial protection against ischemic injury through TMBIM6-VDAC1-mediated regulation of calcium homeostasis and mitochondrial quality surveillance. Phytomedicine. 132:155331. doi: 10.1016/j.phymed.2023.155331. 38870748

[CIT0007] Chang X, Zhou S, Liu J, Wang Y, Guan X, Wu Q, Zhang Q, Liu Z, Liu R. 2024. Zishen Tongyang Huoxue decoction (TYHX) alleviates sinoatrial node cell ischemia/reperfusion injury by directing mitochondrial quality control via the VDAC1-β-tubulin signaling axis. J Ethnopharmacol. 320:117371. doi: 10.1016/j.jep.2023.117371. 37981118

[CIT0008] Chen Y, Chu J, Jiang Z, Gao Z. 2024. Effect of Qingxin Jieyu Granules on mitophagy regulation and ventricular remodeling after myocardial infarction in C57B/L6 mice. Chin J Exp Tradit Med Formulae.[accessed 2024 Sep 20]:[13 p.]. doi: 10.13422/j.cnki.syfjx.20241436. Chinese

[CIT0009] Cheng J, Ren C, Cheng R, Li Y, Liu P, Wang W, Liu L. 2021. Mangiferin ameliorates cardiac fibrosis in D-galactose-induced aging rats by inhibiting TGF-β/p38/MK2 signaling pathway. Korean J Physiol Pharmacol. 25(2):131–137. doi: 10.4196/kjpp.2021.25.2.131. 33602883 PMC7893489

[CIT0010] Dewald O, Zymek P, Winkelmann K, Koerting A, Ren G, Abou-Khamis T, Michael LH, Rollins BJ, Entman ML, Frangogiannis NG. 2005. CCL2/monocyte chemoattractant protein-1 regulates inflammatory responses critical to healing myocardial infarcts. Circ Res. 96(8):881–889. doi: 10.1161/01.RES.0000163017.13772.3a. 15774854

[CIT0012] Frangogiannis NG. 2012. Regulation of the inflammatory response in cardiac repair. Circ Res. 110(1):159–173. doi: 10.1161/CIRCRESAHA.111.243162. 22223212 PMC3690135

[CIT7140409] Frangogiannis NG. 2014. The inflammatory response in myocardial injury, repair, and remodelling. Nat Rev Cardiol. 11(5):255–265. doi: 10.1038/nrcardio.2014.28. 24663091 PMC4407144

[CIT0013] Frangogiannis NG. 2024. TGF-β as a therapeutic target in the infarcted and failing heart: cellular mechanisms, challenges, and opportunities. Expert Opin Ther Targets. 28(1-2):45–56. doi: 10.1080/14728222.2024.2316735. 38329809

[CIT0014] Gao X. 2017. Clinical study of Qing-Xin-Jie-Yu Fang intervention in stable coronary heart disease patients with enhanced inflammatory response. [Master’s thesis]. Beijing: Chinese Academy of Chinese Medicine.

[CIT0015] Gaur R, Mensah KA, Stricker J, Adams M, Parton A, Cedzik D, Connarn J, Thomas M, Horan G, Schafer P, et al. 2022. CC-99677, a novel, oral, selective covalent MK2 inhibitor, sustainably reduces pro-inflammatory cytokine production. Arthritis Res Ther. 24(1):199. doi: 10.1186/s13075-022-02850-6. 35982464 PMC9386913

[CIT0016] Hitti E, Iakovleva T, Brook M, Deppenmeier S, Gruber AD, Radzioch D, Clark AR, Blackshear PJ, Kotlyarov A, Gaestel M. 2006. Mitogen-activated protein kinase-activated protein kinase 2 regulates tumor necrosis factor mRNA stability and translation mainly by altering tristetraprolin expression, stability, and binding to adenine/uridine-rich element. Mol Cell Biol. 26(6):2399–2407. doi: 10.1128/MCB.26.6.2399-2407.2006. 16508014 PMC1430282

[CIT0017] Jiang Z. 2022. Regulation of mitochondrial autophagy by Qing-Xin-Jie-Yu Fang granules improves ventricular remodelling after myocardial infarction. [Dissertation]. Beijing: Chinese Academy of Chinese Medicine.

[CIT0018] Ju J. 2019. Mechanism study on stabilisation of atherosclerotic vulnerable plaques by regulating macrophage pyroptosis with Qing-Xin-Jie-Yu Fang prescription. [Dissertation]. Beijing: Beijing University of Traditional Chinese Medicine.

[CIT0019] Ke D, Ni J, Yuan Y, Cao M, Chen S, Zhou H. 2024. Identification and validation of hub genes related to neutrophil extracellular traps-mediated cell damage during myocardial infarction. J Inflamm Res. 17:617–637. doi: 10.2147/JIR.S444975. 38323113 PMC10844013

[CIT0020] Kologrivova I, Shtatolkina M, Suslova T, Ryabov V. 2021. Cells of the immune system in cardiac remodeling: main players in resolution of inflammation and repair after myocardial infarction. Front Immunol. 12:664457. doi: 10.3389/fimmu.2021.664457. 33868315 PMC8050340

[CIT0021] Lee TH, Hamilton MA, Stevenson LW, Moriguchi JD, Fonarow GC, Child JS, Laks H, Walden JA. 1993. Impact of left ventricular cavity size on survival in advanced heart failure. Am J Cardiol. 72(9):672–676. doi: 10.1016/0002-9149(93)90883-e. 8249843

[CIT0022] Lei W, Li X, Li L, Huang M, Cao Y, Sun X, Jiang M, Zhang B, Zhang H. 2021. Compound Danshen dripping pill ameliorates post ischemic myocardial inflammation through synergistically regulating MAPK, PI3K/AKT and PPAR signaling pathways. J Ethnopharmacol. 281:114438. doi: 10.1016/j.jep.2021.114438. 34390798

[CIT0023] Li J, Gao Z, Zhang L, Li S, Yang Q, Shang Q, Gao X, Qu H, Gao J, Shi L, et al. 2019. Qing-Xin-Jie-Yu granule for patients with stable coronary artery disease (QUEST Trial): a multicenter, double-blinded, randomized trial. Complement Ther Med. 47:102209. doi: 10.1016/j.ctim.2019.102209. 31780034

[CIT0024] Li Z, Lin C, Cai X, Hu S, Lv F, Yang W, Zhu X, Ji L. 2023. Anti-inflammatory therapies were associated with reduced risk of myocardial infarction in patients with established cardiovascular disease or high cardiovascular risks: a systematic review and meta-analysis of randomized controlled trials. Atherosclerosis. 379:117181. doi: 10.1016/j.atherosclerosis.2023.06.972. 37527612

[CIT0025] Liu W, Cheng L, Li X, Zhao L, Hu X, Ma Z. 2022. Short-term pretreatment of naringin isolated from Citrus wilsonii Tanaka attenuates rat myocardial ischemia/reperfusion injury. Naunyn Schmiedebergs Arch Pharmacol. 395(9):1047–1059. doi: 10.1007/s00210-022-02255-x. 35666279

[CIT0026] Li J. 2018. A randomised double-blind controlled study of the effect of Qing-Xin-Jie-Yu Fang on clinical endpoint events in stable coronary heart disease. [Dissertation]. Beijing: Beijing University of Chinese Medicine.

[CIT0028] Lv B, Liu X, Tan W, Wang X, Gao X. 2021. Study of the role of the inflammatory cascade response in the development of myocardial infarction and its pharmacological treatment. J Tianjin Univ Tradit Chin Med. 40(04):424–430. [Chinese].

[CIT0029] Mahtani KR, Brook M, Dean JL, Sully G, Saklatvala J, Clark AR. 2001. Mitogen-activated protein kinase p38 controls the expression and posttranslational modification of tristetraprolin, a regulator of tumor necrosis factor alpha mRNA stability. Mol Cell Biol. 21(19):6461–6469. doi: 10.1128/MCB.21.9.6461-6469.2001. 11533235 PMC99793

[CIT0030] Matter MA, Paneni F, Libby P, Frantz S, Stähli BE, Templin C, Mengozzi A, Wang YJ, Kündig TM, Räber L, et al. 2024. Inflammation in acute myocardial infarction: the good, the bad and the ugly. Eur Heart J. 45(2):89–103. doi: 10.1093/eurheartj/ehad486. 37587550 PMC10771378

[CIT0031] Nakano A, Baines CP, Kim SO, Pelech SL, Downey JM, Cohen MV, Critz SD. 2000. Ischemic preconditioning activates MAPKAPK2 in the isolated rabbit heart: evidence for involvement of p38 MAPK. Circ Res. 86(2):144–151. doi: 10.1161/01.res.86.2.144. 10666409

[CIT0032] Pan W, Zhou G, Hu M, Li G, Zhang M, Yang H, Li K, Li J, Liu T, Wang Y, et al. 2024. Coenzyme Q10 mitigates macrophage mediated inflammation in heart following myocardial infarction via the NLRP3/IL1β pathway. BMC Cardiovasc Disord. 24(1):76. doi: 10.1186/s12872-024-03729-x. 38281937 PMC10822151

[CIT0033] Prabhala P, Ammit AJ. 2015. Tristetraprolin and its role in regulation of airway inflammation. Mol Pharmacol. 87(4):629–638. doi: 10.1124/mol.114.095984. 25429052

[CIT0034] Pradeep SR, Thirunavukkarasu M, Accorsi D, Swaminathan S, Lim ST, Cernuda B, Kemerley A, Hubbard J, Campbell J, Wilson RL, et al. 2024. Novel approaches to determine the functional role of cardiomyocyte specific E3 ligase, Pellino-1 following myocardial infarction. Biochim Biophys Acta Mol Basis Dis. 1870(1):166899. doi: 10.1016/j.bbadis.2023.166899. 37778482

[CIT0035] Quan J, Yang L, Deng X, Wang C, Kong Y, Chen P, Peng J, Wang X, Du T, Chen C, et al. 2023. Rapid identification of components in Qingxin Jieyu Granules using UHPLC-Q-Exactive Orbitrap MS and molecular network technology. Chin J Tradit Chin Med Pharm. 38(12):5709–5722. [Chinese].

[CIT0036] Reboll MR, Korf-Klingebiel M, Klede S, Polten F, Brinkmann E, Reimann I, Schönfeld HJ, Bobadilla M, Faix J, Kensah G, et al. 2017. EMC10 (endoplasmic reticulum membrane protein complex subunit 10) is a bone marrow-derived angiogenic growth factor promoting tissue repair after myocardial infarction. Circulation. 136(19):1809–1823. doi: 10.1161/CIRCULATIONAHA.117.029980. 28931551

[CIT0037] Rezcallah MC, Al-Mazi T, Ammit AJ. 2021. Cataloguing the phosphorylation sites of tristetraprolin (TTP): functional implications for inflammatory diseases. Cell Signal. 78:109868. doi: 10.1016/j.cellsig.2020.109868. 33276085

[CIT3896167] Ronkina N, Menon MB, Schwermann J, Tiedje C, Hitti E, Kotlyarov A, Gaestel M. 2010. MAPKAP kinases MK2 and MK3 in inflammation: complex regulation of TNF biosynthesis via expression and phosphorylation of tristetraprolin. Biochem Pharmacol. 80(12):1915–1920. doi: 10.1016/j.bcp.2010.06.021. 20599781

[CIT0038] Sandler H, Kreth J, Timmers HT, Stoecklin G. 2011. Not1 mediates recruitment of the deadenylase Caf1 to mRNAs targeted for degradation by tristetraprolin. Nucleic Acids Res. 39(10):4373–4386. doi: 10.1093/nar/gkr011. 21278420 PMC3105394

[CIT0039] Shen Z, Shen A, Chen X, Wu X, Chu J, Cheng Y, Peng M, Chen Y, Weygant N, Wu M, et al. 2020. Huoxin pill attenuates myocardial infarction-induced apoptosis and fibrosis via suppression of p53 and TGF-β1/Smad2/3 pathways. Biomed Pharmacother. 130:110618. doi: 10.1016/j.biopha.2020.110618. 34321167

[CIT0040] Solomon SD, Skali H, Anavekar NS, Bourgoun M, Barvik S, Ghali JK, Warnica JW, Khrakovskaya M, Arnold JM, Schwartz Y, et al. 2005. Changes in ventricular size and function in patients treated with valsartan, captopril, or both after myocardial infarction. Circulation. 111(25):3411–3419. doi: 10.1161/CIRCULATIONAHA.104.508093. 15967846

[CIT0042] Stoecklin G, Stubbs T, Kedersha N, Wax S, Rigby WF, Blackwell TK, Anderson P. 2004. MK2-induced tristetraprolin:14-3-3 complexes prevent stress granule association and ARE-mRNA decay. Embo J. 23(6):1313–1324. doi: 10.1038/sj.emboj.7600163. 15014438 PMC381421

[CIT0043] Streicher JM, Ren S, Herschman H, Wang Y. 2010. MAPK-activated protein kinase-2 in cardiac hypertrophy and cyclooxygenase-2 regulation in heart. Circ Res. 106(8):1434–1443. doi: 10.1161/CIRCRESAHA.109.213199. 20339119 PMC2903446

[CIT0044] Taylor GA, Carballo E, Lee DM, Lai WS, Thompson MJ, Patel DD, Schenkman DI, Gilkeson GS, Broxmeyer HE, Haynes BF, et al. 1996. A pathogenetic role for TNF alpha in the syndrome of cachexia, arthritis, and autoimmunity resulting from tristetraprolin (TTP) deficiency. Immunity. 4(5):445–454. doi: 10.1016/s1074-7613(00)80411-2. 8630730

[CIT0045] Tiedje C, Diaz-Muñoz MD, Trulley P, Ahlfors H, Laaß K, Blackshear PJ, Turner M, Gaestel M. 2016. The RNA-binding protein TTP is a global post-transcriptional regulator of feedback control in inflammation. Nucleic Acids Res. 44(15):7418–7440. doi: 10.1093/nar/gkw474. 27220464 PMC5009735

[CIT0046] Tiedje C, Ronkina N, Tehrani M, Dhamija S, Laass K, Holtmann H, Kotlyarov A, Gaestel M. 2012. The p38/MK2-driven exchange between tristetraprolin and HuR regulates AU-rich element-dependent translation. PLoS Genet. 8(9):e1002977. doi: 10.1371/journal.pgen.1002977. 23028373 PMC3459988

[CIT0047] Wang X, Guo Z, Ding Z, Mehta JL. 2018. Inflammation, autophagy, and apoptosis after myocardial infarction. J Am Heart Assoc. 7(9):e008024. doi: 10.1161/JAHA.117.008024. PMC6015297 29680826

[CIT0048] Wang H, Yang J, Cai Y, Zhao Y. 2024. Macrophages suppress cardiac reprogramming of fibroblasts in vivo via IFN-mediated intercellular self-stimulating circuit. Protein Cell. 15(12):pwae013–929. doi: 10.1093/procel/pwae013. PMC11637486 38530808

[CIT0049] Wang Q, Yang K, Han B, Sheng B, Yin J, Pu A, Li L, Sun L, Yu M, Qiu Y, et al. 2018. Aryl hydrocarbon receptor inhibits inflammation in DSS‑induced colitis via the MK2/p‑MK2/TTP pathway. Int J Mol Med. 41(2):868–876. doi: 10.3892/ijmm.2017.3262. 29207040 PMC5752189

[CIT0050] Wang A. 2019. Remodelling of intestinal flora to regulate lipid metabolism in atherosclerosis by Qingxin Xieyiudu Formula and the mechanism of regulation. [Dissertation]. Beijing: Beijing university of traditional Chinese medicine.

[CIT0051] World Heart Report. 2023. Confronting the World’s Number One Killer. 2023. Geneva, Switzerland: World Heart Federation.

[CIT0052] Wu W, Li D, Zong Y, Zhu H, Pan D, Xu T, Wang T, Wang T. 2013. Luteolin inhibits inflammatory responses via p38/MK2/TTP-mediated mRNA stability. Molecules. 18(7):8083–8094. doi: 10.3390/molecules18078083. 23839113 PMC6270260

[CIT0053] Xiong W, Feng S, Wang H, Qing S, Yang Y, Zhao Y, Zeng Z, Gong J. 2021. Identification of candidate genes and pathways in limonin-mediated cardiac repair after myocardial infarction. Biomed Pharmacother. 142:112088. doi: 10.1016/j.biopha.2021.112088. 34470729

[CIT0054] Xu H, Shi D, Yin H, Zhang J, Chen K. 2008. Blood-stasis and toxin causing catastrophe hypothesis and acute cardiovascular events: proposal of the hypothesis and its clinical significance. Chin J Integr Med. 10:934–938. [Chinese]. 19123336

[CIT0055] Zhai P, Chen Q, Wang X, Ouyang X, Yang M, Dong Y, Li J, Li Y, Luo S, Liu Y, et al. 2024. The combination of Tanshinone IIA and Astragaloside IV attenuates myocardial ischemia-reperfusion injury by inhibiting the STING pathway. Chin Med. 19(1):34. doi: 10.1186/s13020-024-00908-y. 38419127 PMC10900662

