# Supplementary material for: Qing-Xin-Jie-Yu Granule attenuates myocardial infarction-induced inflammatory response by regulating the MK2/TTP pathway
Source: Pharm Biol. 2025 Feb 21;63(1):128–40. doi: 10.1080/13880209.2025.2467377 (PMC11849043; doi:10.1080/13880209.2025.2467377)
Supplement: supplementary material.docx [file IPHB_A_2467377_SM0201.docx]

**Supplementary Table** **1** Information about the antibodies

| Species | Name | Sequence | Dilution | RRID | Catalogue Number | Supplier |
| --- | --- | --- | --- | --- | --- | --- |
| Rabbit | Phospho-MAPKAPK2 (Thr221/Thr222) Antibody | / | 1:1000 | AB_2843749 | AF7309 | Affinity Biosciences |
| Rabbit | MAPKAPK-2 Antibody | / | 1:1000 | AB_10694238 | #3042 | Cell Signaling Technology |
| Mouse | ZFP36 Monoclonal Antibody (OTI1A2) | / | 1:1000 | AB_ 2725702 | MA5-25406 | Thermo Fisher Scientific |
| Rabbit | Vinculin antibody [N1N3] | / | 1:1000 | AB_10732545 | GTX113294 | GeneTex |
| Goat | Goat Anti-Rabbit lgG Antibody,HRP conjugated | / | 1:10000 | AB_895483 | L3012 | SAB |
| Goat | Goat Anti-Mouse IgA Antibody, HRP conjugated | / | 1:10000 | AB_ 3665961 | L35665 | SAB |

**Supplementary Table** **2** Suppliers of materials and reagents with catalog numbers

| Materials and reagents | Catalogue Number | Supplier |
| --- | --- | --- |
| Qing-Xin-Jie-Yu Granule | 2112308 | Jiangyin Tianjiang Pharmaceutical |
| Isosorbide Mononitrate | HY-B0642 | MedChemExpress |
| PBS | PYG0021 | BOSTER Biological Technology |
| DMEM | B1101-001 | BIOEXPLORER Life Sciences |
| Mouse Creatine Kinase Isoenzyme ELISA Kit | MM-43703M1 | Jiangsu Enzyme Immuno Industrial |
| Mouse Brain Natriuretic Peptide/Brain Natriuretic Peptide ELISA Kit | MM-0060M1 | Jiangsu Enzyme Immuno Industrial |
| Mouse Troponin T ELISA Kit | MM-44145M1 | Jiangsu Enzyme Immuno Industrial |
| **H&E stain** | G1120 | Solarbio Life Science |
| Masson's Trichrome stain | G1340 | Solarbio Life Science |
| Bio-Plex Pro Mouse Chemokine assay | M60009RDPD | Bio-Rad |
| CCK-8 reagent | KTC011001 | Abbkine |
| Western & IP Lysis Buffer | P0013 | Beyotime |
| Cocktail | HY-K0010 | MedChemExpress |
| PhosSTOP | 04906845001 | Roche Diagnostics GmbH |
| PMSF | P0100-10 | Solarbio Life Science |
| PVDF membrane | 10600029 | GE Healthcare |
| rapid protein-free solution | PS108P | Epizyme Biotech |
| ECL detection kit | 32109 | Thermo Fisher Scientific |
| Total RNA Extraction Kit | DP451 | Tiangen Biochemical Technology |
| One-Step Reverse Transcription Kit | KR118 | Tiangen Biochemical Technology |
| Fluorescent Quantitative Detection Kit | FP205 | Tiangen Biochemical Technology |

**Chemical analysis of Qing-Xin-Jie-Yu Granule**

The characteristic fingerprint of Qing-Xin-Jie-Yu Granule (QXJYG) was developed by Jiangyin Tianjiang Pharmaceutical Co., Ltd. (Jiangyin, China) using ultra-performance liquid chromatography (UPLC). The UPLC technology was carried out by Thermo Vanquish (Thermo Fisher Scientific Inc., USA), and the separation was performed on an Acclaim RSLC 120 C18 column (2.1×100 mm, 2.2 μm). For UPLC analysis, the injection volume was 1-μl test sample, the flow rate was 0.3 ml/min, and the detection wavelength was 320 nm. In gradient elution using acetonitrile as mobile phase A, and 0.05% phosphoric acid solution containing 10% methanol (adding 0.1g sodium dodecyl sulfate per 100mL) as mobile phase B. QXJYG (0.5g) and chemical standards were dissolved in 80% (w/w) methanol and were processed with ultrasonic and filtration before use.

The retention times and peak area ratios of 18 characteristic peaks on the UPLC fingerprint were investigated as important parameters for the quality control of QXJYG. By comparison with chemical standards, 12 of the 18 characteristic peaks were identified as chlorogenic acid (peak 1), cryptochlorogenic acid (peak 2), magnoflorine (peak 3), calycosin-7-O-β-D-glucoside (peak 6), ferulic acid (peak 7), epiberberine (peak 8), Jatrorrhizine hydrochloride (peak 9), coptisine chloride (peak 10), ononin (peak 13), salvianolic acid (peak 14), berberine (peak 16), and calycosin (peak 18), respectively. A representative chromatogram of tested samples is shown in Supplementary Figure 1 (Chen et al, 2021).

**Quantification of major ingredients in Qing-Xin-Jie-Yu Granule**

In previous research, Anlu Wang (Wang et al, 2022) utilized different UPLC columns to analyze specific ingredients in QXJYG: an ACQUITY UPLC^®^ BEH C18 column for salvianolic acid B and ferulic acid, a Hypersil GOLD C18 column for astragaloside IV, and an Acclaim RSLC 120 C18 column for alkaloids. The separation was achieved using distinct mobile phases: acetonitrile-water (32:68, v/v) for astragaloside IV, acetonitrile with 0.1% phosphoric acid for salvianolic acid B and ferulic acid, and acetonitrile with potassium dihydrogen phosphate for alkaloids. The content of the major ingredients is presented in Supplementary Table 3.


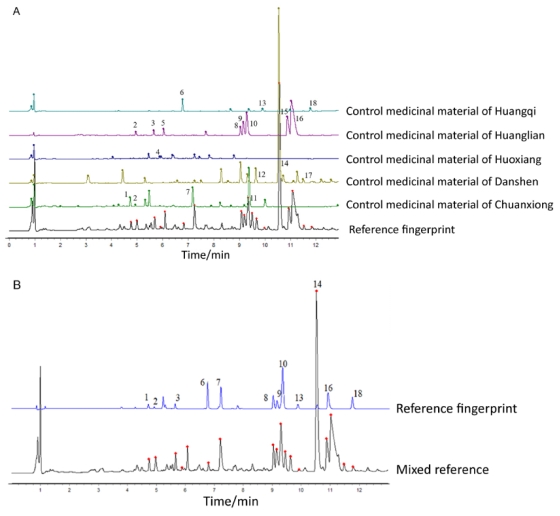


**Supplementary Figure** **1**. A fingerprint of Qing-Xin-Jie-Yu Granule.

**Supplementary Table 3** Quantification of major ingredients in Qing-Xin-Jie-Yu Granule

| Major ingredients | Content (mg/g) |
| --- | --- |
| Salvianolic acid B | 9.9841 |
| Ferulic acid | 0.27 |
| Astragaloside IV | 0.6782 |
| Berberine | 2.2287 |
| Epberberine, coptisine, and palmatine (total amount) | 1.4481 |

**Reference**
Li M, Chen S, Wang X, Jiang Z, Li L, Xu Y, Di L, Gao, Z, 2021. Study on determination of multi-index components and UPLC fingerprint of standard decoction of Qingxin Jieyu Formula. J Nanjing Univ Tradit Chin Med 37,419-427.

Wang A, Guan B, Shao C, Zhao L, Li Q, Hao H, Gao Z, Chen K, Hou Y, Xu H, 2022. Qing-Xin-Jie-Yu Granule alleviates atherosclerosis by reshaping gut microbiota and metabolic homeostasis of ApoE-/- mice. Phytomedicine*.* 2022; 103:154220.
